# Melt electrowriting onto anatomically relevant biodegradable substrates: Resurfacing a diarthrodial joint

**DOI:** 10.1016/j.matdes.2020.109025

**Published:** 2020-08-04

**Authors:** Quentin C. Peiffer, Mylène de Ruijter, Joost van Duijn, Denis Crottet, Ernst Dominic, Jos Malda, Miguel Castilho

**Affiliations:** a Department of Orthopaedics, University Medical Center Utrecht, Utrecht University, GA, Utrecht, the Netherlands; b Regenerative Medicine Center Utrecht, Utrecht, the Netherlands; c RegenHU Ltd, Villaz-St-Pierre, Switzerland; d Department of Clinical Sciences, Faculty of Veterinary Sciences, Utrecht University, Utrecht, the Netherlands; e Orthopaedic Biomechanics, Department of Biomedical Engineering, Eindhoven University of Technology, Eindhoven, the Netherlands

**Keywords:** Fibre-reinforced hydrogels, Anatomical surfaces, Electrostatics, Electrospinning, Osteochondral defects, Biofabrication

## Abstract

Three-dimensional printed hydrogel constructs with well-organized melt electrowritten (MEW) fibrereinforcing scaffolds have been demonstrated as a promising regenerative approach to treat small cartilage defects. Here, we investige how to translate the fabrication of small fibre-reinforced structures on flat surfaces to anatomically relevant structures. In particular, the accurate deposition of MEW-fibres onto curved surfaces of conductive and non-conductive regenerative biomaterials is studied. This study reveals that clinically relevant materials with low conductivities are compatible with resurfacing with organized MEW fibres. Importantly, accurate patterning on non-flat surfaces was successfully shown, provided that a constant electrical field strength and an electrical force normal to the substrate material is maintained. Furthermore, the application of resurfacing the geometry of the medial human femoral condyle is confirmed by the fabrication of a personalised osteochondral implant. The implant composed of an articular cartilage-resident chondroprogenitor cells (ACPCs)-laden hydrogel reinforced with a well-organized MEW scaffold retained its personalised shape, improved its compressive properties and supported neocartilage formation after 28 days in vitro culture. Overall, this study establishes the groundwork for translatingMEWfrom planar and non-resorbable material substrates to anatomically relevant geometries and regenerative materials that the regenerative medicine field aims to create.

## 1 Introduction

Articular cartilage in diarthrodial joints functions as a load-bearing tissue with a nearly frictionless surface. This unique characteristic is at-tributed to the composition of cartilage tissue where its main (structural) components, type II collagen fibrils and glycosaminoglycans (GAGs), as well as cells, are hierarchically distributed throughout the tissue [1–3]. Damage to articular cartilage can cause pain and immobility, and if left untreated, can potentially lead to osteoarthritis (OA) [4,5]. Surgical treatment options for osteochondral defects, such as bone marrow stimulation (for small defects ≤2 cm^2^) [6] and osteochondral grafting [7], or for chondral defects such as cell-based therapies, including autologous chondrocyte implantation (for medium size defects, 2 cm^2^ to 4 cm^2^) [8], are sub-optimal as these typically result in the formation of mechanically weak fibrocartilage tissue [6]. As a last resort option to reduce clinical symptoms and improve patient’s mobility, total knee replacement (TKR) surgery, with the use of metallic implants, is used.[9][9] As the metallic TKR have a limited life-span, this last resort option has to be postponed or even eliminated.[9]

Regenerative approaches based on biofabrication [10] technologies are a potential alternative to repair damaged articular cartilage tissue [11]. Advances in (micro) fibre formation and deposition technologies, such as melt electrowriting (MEW) [12–15] and extrusion-based deposition of bioinks [16], have recently enabled the fabrication of mechanically stable, fibre reinforced cartilage implants [17–19]. Recent developments in the convergence of MEW and extrusion-based technologies within a single manufacturing process allowed the fabrication of constructs with regional compositional variations in both the cellular and fibre components, inspired by what is observed in healthy native articular cartilage [16,20,21]. In particular, this technology-convergence has shown promising results for the fabrication of implants to treat small cartilage defects with coplanar surfaces [16,18]. However, generating human-scale constructs with anatomical relevant shapes still remains a major challenge. The underlying limitation is pre-dominantly related to the electrohydrodynamic working principle of MEW. While MEW relies on the use of a constant electrostatic force to deposit micrometer size fibres in well-organized three dimensional (3D) patterns [22,23], MEW structures are generally deposited onto flat substrates as to not interfere with this electrostatic force.

It is known that the electrical field (EF), and its resulting electrical force, the main fibre pulling force in the MEW process, is affected by the collector design in both shape, dimension [24], and material properties [25], as well as by the instrument configuration and process parameters [26–28]. In particular, the electrical conductive properties of the collecting material, together with the processing parameters, *i.e*. applied voltage, collector-to-spinneret distance, collector velocity, and environmental conditions, are important parameters that can influence accurate MEW fibre deposition [28–30]. While using glass-slides to easily collect fibres [31], only more traditional metallic substrates, such as copper, stainless steel or aluminium, with planar surfaces, have shown to ensure uniform fibre diameters and accurate fabrication of ordered three-dimensional (3D) fibrous microstructures. Unfortunately, most biologically degradable materials used in regenerative medicine are intrinsically nonconductive and native living tissue structures are generally non-flat. For example, materials used for (osteo)chondral repair, such as degradable thermoplastics [32], hydrogels [33,34], or bioceramics [35], are expected to interfere with the EF of the MEW process, while complete diarthrodial joint resurfacing requires accurate patterning, following the anatomical curvature of this joint. To exploit MEW as a technique to produce reinforcing fibres in/onto anatomical relevant shapes and onto clinically relevant materials, it is fundamental to further understand the effects of the electrical properties of the collecting material and its respective geometry on accurate fibre deposition.

**Fig. 1.**
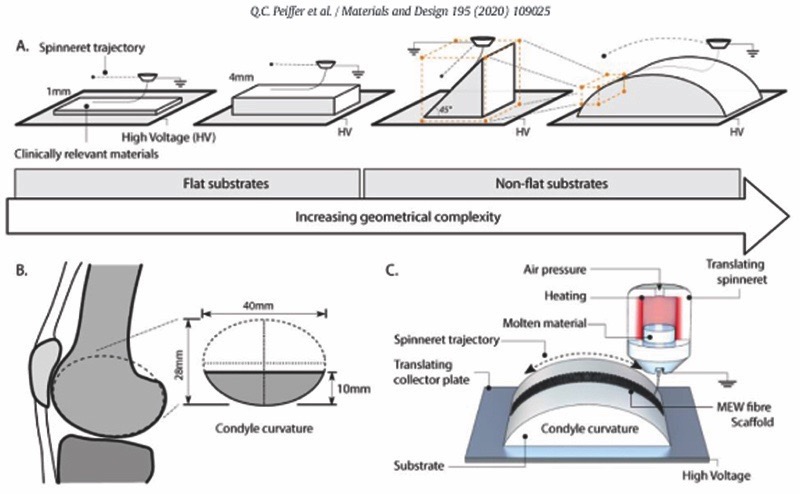
Deposition of melt electrowritten (MEW) fibres on clinically relevant shapes and materials. A) Schematic representation of the different collecting geometries ranging from flat (with a thickness of 1 and 4 mm) to a 45°-wedge and curved dome-shaped structures. B) Curved shape structures were designed to approach the geometry of an average human femoral condyle surface. C) Schematic representation of the MEW process, where PCL micro-fibres are patterned on a substrate with the geometry that mimics the contour of an articulating joint.

Therefore, in this study, we investigate how to translate the fabrication of fibre reinforced structures from flat to more anatomically relevant, non-flat surfaces with the convergence of MEW and extrusion-based printing technologies. In particular, the accurate deposition of MEW-fibres onto anatomically relevant shapes (wedges and curved domes) and biomaterials, *i.e*. bioceramics, magnesium phosphate cement (MgP); thermoplastics, polycaprolactone (PCL); and hydrogels, gelatin methacryloyl (gelMA), is studied (Fig. 1). Through computational modelling, the effect of collecting substrate electrical properties and geometry on the underlying EF distribution and electrical force is investigated. Lastly, the feasibility of fabricating a mechanically reinforced condyle-shaped construct, with biodegradable materials, through the combination of MEW and the extrusion of a cell-laden hydrogel is assessed.

## 2 Materials and methods

### 2.1 Materials

Gelatin methacryloyl (gelMA) was synthesized as previously described [36]. Briefly, gelatin (type A, derived from porcine skin, 175 Bloom, Sigma Aldrich) was dissolved at 10% w/v in phosphate-buffered-saline (PBS) at 60 °C after which 0.6 g methacrylic anhydride (Sigma Aldrich) was added per g of gelatin to achieve an 80% degree of functionalisation. Freeze-dried gelMA was diluted with PBS to obtain a final gelMA concentration of 10% w/v. To initiate the cross-linking reaction, a combination of 5 mM sodium persulfate (Sigma Aldrich) and 0.5 mM Tris(2,2′-bipyridyl) dichlororuthenium (II) hexahydrate (Sigma Aldrich) was added to the gelMA solution and subsequently cast in custom-made PDMS moulds with different shapes (flat/ wedge/dome) and cross-linked for 10 min under led light (20 W LED, Jobmate). Magnesium Phosphate (MgP) was prepared by mixing an Mg_3_(PO_4_) and MgO powder to a 4:1 weight ratio, after which a 3.5% *w/v* polyethylene oxide solution (Mw 600,000 to 1,000,000) (Acros Or-ganics) was added. The solution was cast in above mentioned PDMS moulds and dried at 37 °C for 4 h. Finally, the MgP was hardened with a 3.5 M solution of di-Ammonium hydrogen phosphate (Merck) overnight at 37 °C. Polycaprolactone (PCL, Purasorb PC12, Corbion) was molten at 80 °C and cast in PDMS moulds, while Aluminium (Aluminium 51 ST, Salomon's Metalen B·V) wedge/dome-shaped substrates were directly manufactured by a conventional CNC milling device.

### 2.2 Impedance spectroscopy measurements

Dielectric properties of substrate materials were measured with an impedance/gain-phase analyser (1260 Impedance Analyser, Solartron Analytical) with a dielectric interface (1296A Dielectric Interface System, Solartron Analytical, 12962A Sample Holders, Solartron Analytical). Cylinders (*r* = 20 mm) of 1 mm and 4 mm thicknesses of each material (gelMA, MgP, PCL) were tested at room temperature. An alter-nating current (AC) level of 100 mV was set, and the impedance of each substrate was measured with a frequency sweep (1 MHz – 1 Hz, 5 points/decade). The capacitance (*C*) in Farad of each material composition was obtained and the relative permittivity (*ε*_*r*_) was calculated according to,

(1) $$\varepsilon_{r}=\frac{C}{C_{0}}$$

where C_0_ is the capacity of the empty capacitor. In addition, electrical conductivity (σ) in siemens per metre was obtained indirectly by determining first the material resistance (R) with a digital multimeter, and then obtained by,

(2) $$\sigma =\frac{l}{RA}$$

where l terms represent length and A the cross-sectional area of the measured material specimen.

### 2.3 Surface roughness measurements

Surface roughness of substrate materials was measured using a sur-face roughness tester (SJ-400, Mitutoyo Corp.) as detailed in Supple-mentary Methods.

### 2.4 Melt electrowriting (MEW)

MEW was performed with polycaprolactone (PCL, Purasorb PC12, Corbion) molten in a metallic cartridge at 80 °C with an air pressure of 110–125 kPa, 24G nozzle, voltage of 7–11 kV (3D Discovery Evolution, RegenHU). The printhead was either kept at a constant *Z*-coordinate of 6 mm, or was varied following the surface curvature of the collecting substrate (*Z*-correction) in which the distance between the printing head and the collecting substrate was always6mm. For all experiments, the MEW jet was stabilized prior to printing by printing 40 lines which were analysed for deviations in fibre diameter and/or pulsing [37].

### 2.5 Fibre evaluation

Fibre morphology and fibre stacking was evaluated by scanning electronic microscopy (SEM) (Phenom Pro desktop, ThermoFischer Scientific) (Supplementary Fig. 2A). Samples were coated with 6 nm gold using a rotary pumped coater (Q150R, Quorum Technologies). Prior to SEM, each multi-layered construct was cut in liquid nitrogen with a scalpel.

### 2.6 Printing accuracy quantification

Fibre scaffolds were imaged with an upright microscope (Olympus BX430) and subsequently processed with Fiji (version 2.0.0-rc-54/ 1.51 h). A selection of pores in the central region of the scaffold were selected (Supplementary Fig. 2B) after which the pore ration was measured (Supplementary methods).

### 2.7 Finite element analysis

Numerical simulation of the electric field strength and distribution were performed (COMSOL Multiphysics Simulation Software, Version 5.1 COMSOL Inc.). The MEW printhead and collecting substrate geometries (flat, wedge and curved) were designed according to the printer and substrates used. The electric conductivity of the stainless-steel printhead and collector was set to 1.45 × 10^6^ S m^−1^, and of the air volume to 1 × 10^−15^ S m^−1^. Relative permittivity of 2.3 and 2.7 where defined for substrate materials PLC and MgP, respectively. GelMA and Al were defined as conductive materials (electrical conductivities of 1.25 × 10^−3^ S m^−1^ and 3.20 × 10^7^ S m^−1^, respectively). The electric field strength and distribution was simulated by applying a negative potential to the collector 9 kV, while the spinneret was kept at 0 kV (grounded) at a distance of 6 mm for flat substrates; 6 mm and 31 mm for the wedge substrates; and 6 mm and 11 mm for curved substrates. For simplicity, no charge dissipation was considered.

### 2.8 Fabrication and matrix formation of clinically relevant surfaces

A polycaprolactone (PCL) dome structure was resurfaced with MEW fibres and gelatin methacryloyl (gelMA) hydrogel, encapsulated with articular cartilage progenitor cells (ACPCs). A screw driven extrusion-based PCL printing (3D Discovery Evolution, RegenHU) was used to fabricate the dome structure. PCL was heated to 90 °C and extruded with a extrusion rate of 3 rev/min and a translational speed of 4 mm/s, with 40% porosity. Subsequently, 50 layers of MEW PCL fibres were deposited on top of this dome structure with a collector distanceof 6 mm, collector velocity of 20 mm/s, pressure of 110 kPa, and a voltage of 9 kV. During MEW, the spinneret followed the contour of the dome structure, keeping z-distance constant.

### 2.9 Cell culture

Equine derived articular cartilage-resident progenitor cells (ACPCs) were isolated from the metacarpophalangeal joints of skeletally mature equine donors and expanded as previously described [38]. These donors have been donated to science by their owners and procedures were followed according to the guidelines of the Ethical and Animal Welfare body of Utrecht University [38,39]. After expanding, cells were embedded in 10% gelMA (cell density = 20 * 10^6^ /ml), supplemented with tris-bipyridyl-ruthenium (II) hexahydrate (Ru, 0.2 mM, Sigma Aldrich)/so-dium persulfate (SPS, 2 mM, Sigma Aldrich), which was either deposited on top of the MEW structure of the PCL dome with a pipet, or perfused in a Teflon mould to fabricate cell-laden cast 3D discs (height = 2 mm, diameter = 6 mm) that was used a control group. Crosslinking occurred under visible light conditions for 10 min after which the constructs were cultured in chondrogenic differentiation medium (Supplementary methods) for 28 days. All cultures were performed under sterile and normoxic culture conditions at a temperature of 37 °C and 5% CO_2_.

### 2.10 In vitro evaluation of (bio) fabricated implants

During culture, metabolic activity was measured using a resazurin assay (Sigma Aldrich) at day 1,7,14, 28. After 28 days, matrix formation was quantified by biochemical assessment of GAG (dimethylmethylene Blue (DMMB),SigmaAldrich) per DNA (Quant-iT-Picogreen-dsDNA-kit, Invitrogen) according to manufacture protocols. Additionally, samples were embedded in MMA, polymerized, and saw, or paraffin embedded and cut, to visualize the cell distribution (haematoxylin staining (Sigma Aldrich)) and matrix distribution, respectively. Matrix distribution was visualized with a safranin O (Sigma Aldrich), combined with fast green to stain fibrous tissue (Sigma Aldrich). Immunohistochemistry was performed to visualize type II collagen (II-II6B3, DSHB).

### 2.11 Mechanical analysis of fibre reinforced gelMA scaffolds

Uniaxial compression tests were performed on a universal testing machine (Zwick Z010, Germany) equipped with a 20 N load cell. Tests were conducted at a rate of 1 mm/min at room temperature, with all samples immersed in PBS to approximate physiological conditions. Cylindrical samples with a diameter of 5 mm and a height of 1 mm were used. For each engineered stress-strain curve, the elastic modulus, de-fined as the slope of the linear region from 0.15 to 0.20 mm/mm. was determined.

### 2.12 Statistical analysis

Data is represented as mean ± standard deviation. For surface roughness measurements, impedance spectroscopy, fibre diameter measurements, scaffold thickness, pore ratio, and *in vitro* studies, at least 3 samples per group were used. For the mechanical tests, at least 4 samples were analysed per group. An unpaired *t*-test (GAG/DNA) and a two-way ANOVA, *post hoc* Tukey test (metabolic activity) were used to test the difference between the cultured disc and dome structures. A one-way ANOVA with Tukey's post hoc test was used to compare the means of the different groups for the mechanical data, fibre diameter, scaffold thickness, and pore ratio measurements. Test differences were considered significant at a probability error (p) of *p* ˂ .05. Normality and homogeneity were checked with Shapiro-Wilks and Levene's tests, using GraphPad Prism version 6 (San Diego, USA).

## 3 Results

### 3.1 Material properties: Surface roughness and electrical conductive properties

Magnesium phosphate (MgP) and gelMA substrates presented higher surface roughness values (R_a_ and R_q_ between 3.61 and 5.21 μm) than PCL and Aluminium substrates (R_a_ and R_q_ between 0.07 and 0.32 μm) (Supplementary Table 1). Impedance spectroscopy confirmed the electrical conductivity and relative permittivity of PCL, MgP, gelMA, and aluminium (Al) collecting substrates of 1 and 4 mm (Table 1). PCL and MgP behaved as non-conductive materials with relative permittivity values of 2.11 and 4.32, respectively. GelMA was confirmed partly conductive with a relative permittivity of 5 × 10 [7]. Aluminium confirmed its conductive properties as measured relative permittivity was out of the measurement range.

### 3.2 Effect of collecting material conductivity on fibre deposition

**Fig. 2.**
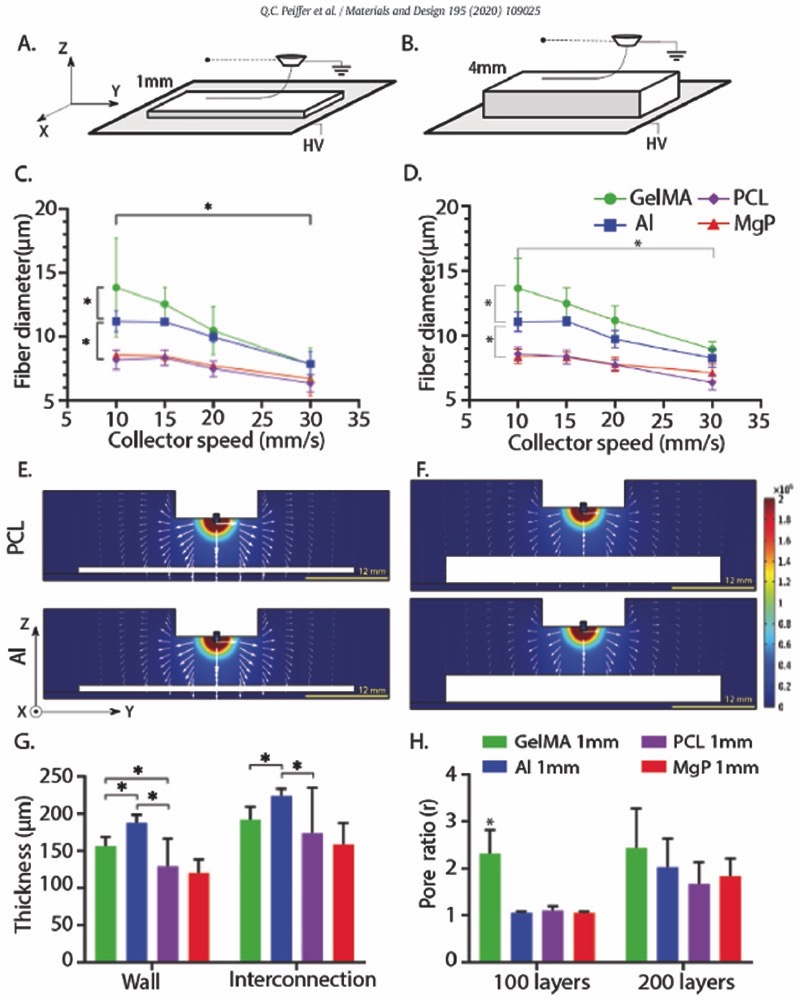
Fibre collection on flat-shaped collecting materials (PCL, MgP, gelMA, and Al). A, B) collecting materials of 1 mm and 4 mm high were investigated. C) Effect of collector velocity on fibre diameter of fibres deposited on 1 mm thick collectors. D) Effect of collector velocity on fibre diameter of fibres deposited on 4 mm thick collectors. E) Computational simulation of EF strength (V/m) and distribution (white arrows in logarithmic scale) for non-conductive (PCL) and conductive (Al) collecting material of E) 1 mm and F) 4 mm thick. G) Final scaffold thickness as a reflection of fibre stacking accuracy. Collector velocity = 15 mm/s. H) Pore ratio of scaffolds deposited on 1 mm thick collectors (*r* = 1 indicates a printed scaffold that conforms to the planned design, while values *r* ˃ 1 indicates imperfect fibre stacking).* = *p* ˂ .05.

The effect of the conductivity of the collecting materials on fibre deposition was studied using substrates with thicknesses of 1 and 4 mm (Fig. 2A, B). A significant smaller fibre diameter was observed when depositing onto non-conductive substrates (8 μm for PCL and MgP) as compared to collecting onto conductive substrates (˃ 11 μm for gelMA and aluminium) (Fig. 2C). This phenomenon was confirmed for different collector velocities (Fig. 2C) and different voltages (Supplementary Fig. 1A). Morphologically, fibres deposited onto PCL and MgP maintained a cylindrical shape (Supplementary Fig. 1B) whereas fibres deposited on aluminium and gelMA were more flattened (ellipsoidal) for the first layer (Supplementary Fig. 1C) this morphology was not directly related to material surface roughness. Collecting material thickness did not affect fibre diameters. Computational simulation confirmed that the EF strength along the *Z*-axis (*i.e*. out-of-collector plane direction) was slightly higher for conductive collecting materials as compared to the non-conductive collecting materials. The profile of the EF along the Z-axis was independent of substrate thickness (Fig. 2E, F). Additionally, the global EF distribution was similar for the different substrate materials and concentrated predominantly around the spinneret.

Scaffolds fabricated onto non-conductive materials (PCL, MgP) were ~ 50 μm less high as compared to scaffolds fabricated onto conductive materials (gelMA, aluminium) (Fig. 2G, Supplementary Fig. 2A). Scaffolds printed on gelMA showed more (2 – fold) deviation from design than the scaffolds printed onto PCL, MgP, or aluminium (Fig. 2H, Supplementary Fig. 2B). Overall, an increase in the amount of layers from 100 to 200 resulted in higher pore ratios, which demonstrated a decrease in fibre stacking accuracy upon increasing the amount of layers. The thickness (1 mm or 4 mm) of the collecting material did not affect accurate fibre stacking (Supplementary Fig. 2C).

### 3.3 Effect of collector geometry on fibre deposition:non-flat, wedge and curved substrates

Printing onto a 45° wedge substrates showed similar trends as printing onto dome-structures. In general, printing on wedge-shaped collecting materials without z-correction in the printing trajectory (Supplementary Fig. 3A), resulted in poor fibre placement (inconsistency in fibre diameter and consequently distorted scaffold architectures) (Supplementary Fig. 3B). Computational simulations showed that the EF strength at the surface of the wedge decreased when printing without a z-correction in printing trajectory (Supplementary Fig. 3C). For prints with a z-corrected trajectory, the EF strength remained constant and the electrical force is normal to wedge surface.

These observations are slightly more pronounced for less conductive materials (PCL and MgP). Scaffolds fabricated (using z-trajectory correction) onto non-conductive wedges (PCL, MgP) were 100 to 200 μm smaller than scaffolds fabricated onto conductive wedges (gelMA, aluminium) (Supplementary Fig. 3C–D). Scaffolds fabricated on PCL and MgP wedges featured two-fold lower pore ratio than scaffolds fabri-cated onto the gelMA wedge.

**Fig. 3.**
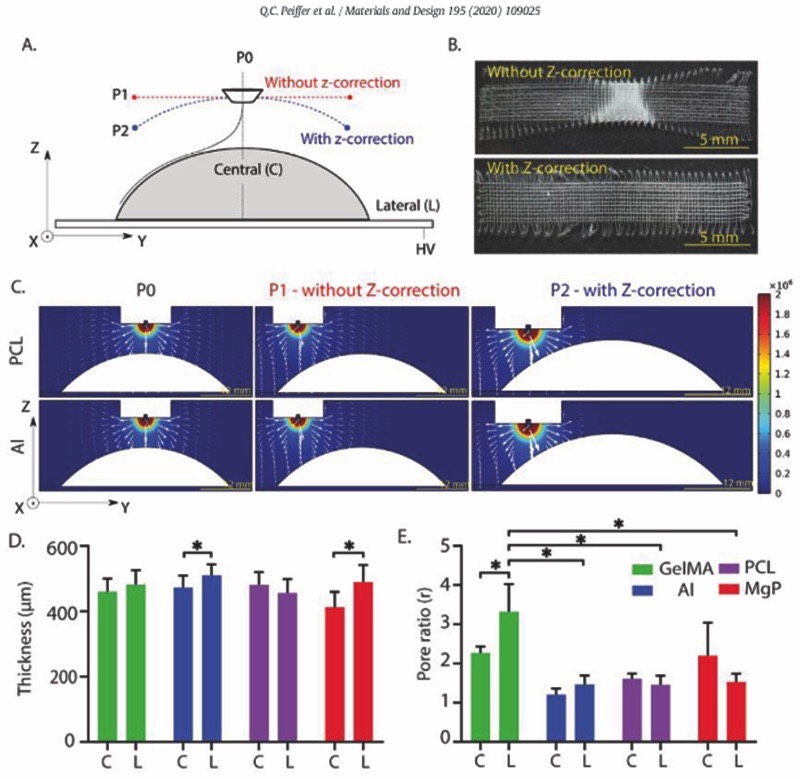
Fibre collection on curved collecting materials (PCL, MgP, gelMA, and Al). A) Schematic representation of the evaluated printhead trajectories with and without z-correction. B) Representative stereoscopic images of scaffolds printed on aluminium dome-shaped structures with and without z-correction of the printhead trajectory. Not significant differences in the fibre diameter between central and lateral dome section were observed. C) Computational simulation of EF strength (V/m) and distribution (white arrows in logarithmic scale) for a non-conductive (PCL) and conductive (aluminium) curved-shaped collecting materials. Quantification of D) the final scaffold thickness and E) the pore ratio of scaffolds deposited on curved-shaped collecting materials with z-correction in the printhead trajectory. C and L represent central and lateral parts of the dome structures, respectively. * = p ˂ .05.

*Z*-correction in the printhead trajectory also improved the accuracy of fibre deposition when printing onto dome-structures (Fig. 3A, B). Computational simulation confirmed that the EF strength was constant and normal to the curved surface when printing with z-correction in the printhead trajectory (Fig. 3C). Although scaffold thickness was relatively unaffected by the collecting material used, scaffold printed on Al and MgP substrates did show a significant difference between the thickness of fibres deposited on top of the dome (centre) or more at the edges (lateral) (Fig. 3D). Additionally, gelMA showed a higher pore ratio compared to PCL, MgP, and aluminium (Fig. 3E). No significant differences were found between the lateral and the central parts of the scaffolds fabricated on the curved-shaped PCL or gelMA, when using a z-correction in the printhead trajectory (Supplementary Fig. 4).

### Resurfacing the entire joint surface – Simple convex surfaces

**Fig. 4.**
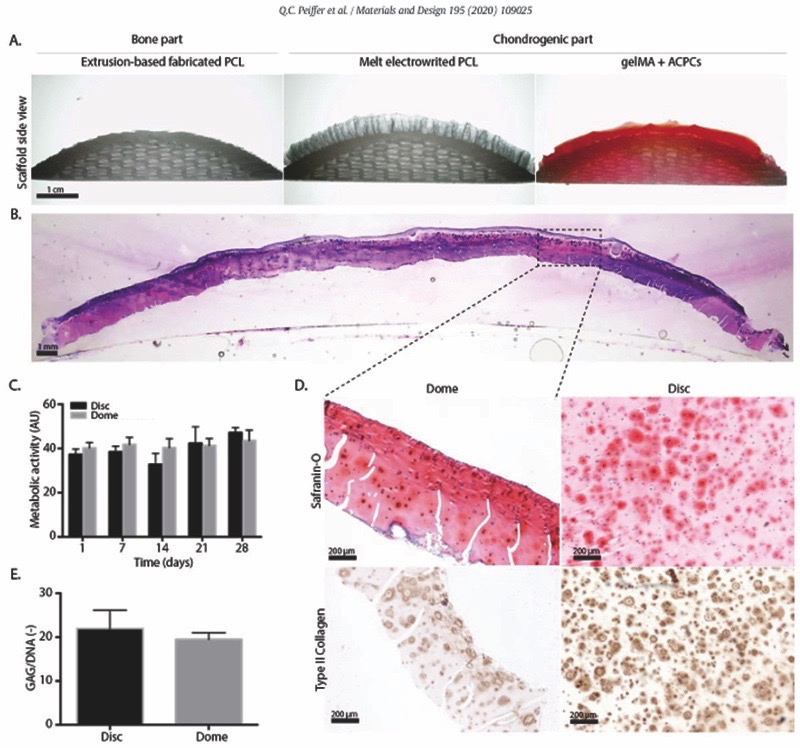
Resurfacing a fully resorbable PCL mimicking contour of a human femoral condyle surface and cartilage-like tissue formation after 28 days of *in vitro* culture. A) Macroscopic cross section of MEW fibre-reinforced gelMA hydrogel with encapsulated articular chondrocyte progenitor cells onto an extruded PCL substrate that approximates native human femur curvature. B) H&E staining of the manufactured implant after culture. C) Metabolic activity of cast discs and printed femoral structures. D) Safranin O and type II collagen staining of printed femoral structures sections after culture. E) Proteoglycan content normalized to DNA.

A PCL scaffold, approximating the native curvature of an average human femur, was successfully resurfaced with a boxed-microfibre architecture and filled with a cell-laden gelMA hydrogel (Fig. 4A). The interfibre spacing (400 μm) that was used in this study to mechanically reinforce the cell-laden hydrogel, indeed showed a significant increase in the compressive modulus as compared to hydrogel only and scaffold only groups (Supplementary Fig. 5). During the 28 days of culture, the fibre reinforced hydrogel on top of the femur structure retained its shape (Fig. 4B) and embedded cells showed comparable metabolic ac-tivity to those in cast disc controls (Fig. 4C). Histological evaluation of the constructs revealed abundant positive staining for safranin O and and type II collagen (Fig. 4D). No significant differences in cartilage-like matrix deposition was observed between the condyle-shaped constructs and the cast disc controls (Fig. 4E).

## 4 Discussion

This study demonstrates the challenges of translating the fabrication of microfibre reinforcing scaffolding structures from flat to curved, anatomically relevant geometries and clinically relevant material with multi-scale bioprinting technologies. Although MEW fibre stacking on gelMA was less accurate as compared to MEW fibre stacking on PCL, MgP, and aluminium, accurate deposition of MEW-fibres onto clinically relevant material and anatomically relevant shapes was achieved. Fur-thermore, a converged printed, resurfaced human condyle-shaped con-struct was fabricated and supported cartilage-tissue formation after 28 days of *in vitro* culture.

It was shown that MEW fibre diameters are strongly affected by the materials they are printed on. Printing on conductive materials (gelMA, aluminium) resulted in larger fibre diameters as compared to printing on non-conductive materials (PCL, MgP). This observation can be explained by the fact that dielectric materials modifying the overall EF strength, which consequently disturbs the orientation and pulling force exerted on the molten jet [25,29,40]. Fibres collected on the PCL substrates had a more cylindrical morphology compared to those col-lected on gelMA. This could be attributed to the more homogeneous fibre-cooling prior the deposition of the fibres as a result of the EF-induced jet-lag. Interestingly, fibre diameter was not affected by the thickness of the collecting materials used, which confirmed that electrical conductive properties are not significantly affected by the dimensions, within the range of the present experiment, of the material. In addition, we observed that the effect of the material composition of the collecting substrates on the deposition of the fibres was less than would be expected based on the previously observed disturbance of the EF by dielectric materials [29,30,40]. However, the scaffolds fabricated on PCL and MgP structures had a high deposition accuracy, as demonstrated by the high pore ratio, and was comparable to the accuracy obtained on aluminium substrates. The permittivity of both PCL and MgP was not high enough to generate significant disturbance of the EF and consequently, affect the accuracy of the fibre depositioning process. Our computational simulations confirm this hypothesis, as only small differences in the EF strength magnitude were determined when using PCL and MgP as collecting materials in comparison to the more conductive collecting materials.

Interestingly, accurate fibre deposition on non-flat, *i.e*. wedge- and curve-shaped, collecting materials was significantly improved when the distance between the printhead and the collecting substrate material remained constant (*i.e*. when applying a z-correction to the printhead trajectory). It has been shown that an increase in collector distance leads to a drop in the overall EF strength if the voltage is not increased accordingly [41]. Consistent with literature that shows the effect of substrate geometry on jet deflection [42], our computational simulations showed a significant change in the global EF distribution when z-correction is employed. This data demonstrates that when applying z-correction to the printhead trajectory, the geometry of the substrate is not a limiting element in accurate fibre deposition on non-flat substrates.

Although we have shown that accurate deposition of MEW fibres is possible on different collecting materials and on anatomically relevant geometries, some deposition inaccuracies were still observed. In general, fibre deposition was less accurate on gelMA as a collecting surface compared to deposition on PCL, MgP, and aluminium substrates. This could possibly be due to the high-water content of gelMA, as evaporation of water due to the proximity of the heated MEW spinneret affects the electrical properties of gelMA during the fabrication process. Additionally, we hypothesized that evaporation of water could potentially result in an increase of the local humidity and, therefore, cause disturbance the EF. Moreover, as gelMA is a soft, viscoelastic material, the wedge and dome collecting structures made of gelMA were more prone tomovements upon the vibrations that were induced by fast machine displacement (due to the fast collecting velocities used), increasing the instabilities during fibre deposition on these substrates. The observed decreased accuracy of fibre deposition with increasing fibre scaffold thickness is consistent with recent literature and described as possibly due to remnant charges trapped in already deposited fibres [41].

As a potential application to treat full-thickness cartilage or osteochondral defects, we demonstrated the fabrication of structures with a fibre-reinforced osteochondral construct that anatomically reflects the curvature of the native femoral condyle surface. Notably, these constructs presented high shape fidelity and remained shape during the 28-day of *in vitro* culture period. Moreover, homogenous and abundant cartilage-like tissue formation throughout the cartilage compartment of the constructs was observed. This demonstrates that reinforcing strategies could be translated from frequently fabricated small, osteochondral plugs with flat, coplanar surfaces [43], towards anatomically relevant structures with patient-specific geometries. Although resurfacing anatomically relevant surfaces has previously been shown with a dense fibre matrix, those scaffolds did not allow for cell deposition and homogeneous extra cellular matrix [44]. To the best of the our knowledge, this is the first study to report the fabrication of a larger, microfibre reinforced, low fibre density, hydrogel-based construct that follow the articulating surface. Ultimately, to investigate the effect of different anatomically relevant geometries and biological applicable material combinations, future studies should consider to evaluate microfibre patterning onto convex and irregular shaped geometries composed of more than one material combination, and with included porosity for the bone-reflecting component.

## 5 Conclusion

Taken together, this study demonstrates the printing of well-organized microfibre scaffolds on clinically relevant collecting materials with non-flat geometries. The electrical properties of the substrate materials revealed a greater impact on accurate fibre deposition than the substrate thickness. Notably, deposition of MEW fibres was possible not only on conductive resorbable materials, like hydrogels, but also on less conductive materials, including bioceramics and thermoplasts. Accurate fibre deposition on non-flat geometries (wedge- and curved-shape structures) was shown to be successful, yet, maintaining the electrostatic force constant and normal to the collecting surface was fundamental for the successful deposition of micro sized fibres. This further understanding of the underlying physical principles of the MEW process allowed the fabrication of a complete condyle-shaped biological construct, for which abundant cartilage-like matrix formation after 28 days of *in vitro* culture was shown. Overall, these findings establish the groundwork for further translation of the convergence of MEW and bioprinting, from flat to anatomical relevant structures, that the regenerative medicine and biofabrication fields aim to recreate.

## Supplementary Material

Supplementary data to this article can be found online at https://doi. org/10.1016/j.matdes.2020.109025.

Supplementary Materials

Supplementary video 1

Supplementary video 2

## Figures and Tables

**Table 1:** Relative permittivity (ε_r_) and electrical conductivity (σ) of investigated materials.

Substrate biomaterial	Relative permittivity (ε_r_, at 1 Hz)	Electrical conductivity (σ, Sm ^−1^)
Polycaprolactone (PCL)	2.11	/
Magnesium phosphate based cement (MgP)	4.32	/
Gelatine methacryloyl (gelMA)	5 × 10^7^	4.17 × 10^−3^–1.25 × 10^−2^
Aluminium (Al, control)	/	3.20 × 10^7^

## Highlights

- Microfibre patterning onto an anatomical relevant structure and clinically relevant materials is demonstrated.
- Accurate microfibre patterning is demonstrated on both electrically conductive and non-conductive materials.
- Fabrication of a microfibre-reinforced, cell-laden hydrogel that resurfaces a human biological joint replica is shown
