# Supplementary material for: Melt electrowriting onto anatomically relevant biodegradable substrates: Resurfacing a diarthrodial joint
Source: Mater Des. Author manuscript; Available in PMC 2020 Oct 20. (PMC7116215; doi:10.1016/j.matdes.2020.109025)
Supplement: Supplementary Materials [file EMS96603-supplement-Supplementary_Materials.docx]

**Title: Melt electrowriting onto anatomically relevant** **biodegradable substrates: Resurfacing a diarthrodial joint**

Quentin C. Peiffer, Mylène de Ruijter, Joost van Duijn, Denis Crottet, Ernst Dominic, Jos Malda, Miguel Castilho

**Supplementary methods**

**Surface roughness measurements:** Surface roughness of substrate materials was measured using a surface roughness tester (SJ-400, Mitutoyo Corp.). Tests were carried out at 0.5 mm/s scanning speed and covered a specimen length of approximately 4 mm. For each substrate material, the average surface roughness ($R_{a}$) and the route mean square route roughness ($R_{q}$) was evaluated. $R_{a}$ was calculated as,

| $R_{a}=\frac{1}{L}\sum_{x=1}^{L} \left\vert Z_{x} \right\vert$ | (3) |
| --- | --- |

where L is the evaluation length of the substrate material and $Z_{x}$ is the measured profile height. $R_{q}$ was obtained by,

| $R_{q}=\sqrt{\frac{1}{L}\sum_{x=1}^{L} Z^{2}x}$ | (4) |
| --- | --- |

All measurements were repeated at least three times and in two different locations of each substrate material.

**Printing accuracy quantification:** Fibre scaffolds were imaged with an upright microscope (Olympus BX430) and subsequently processed with Fiji (version 2.0.0-rc-54/1.51h). A selection of pores in the central region of the scaffold were selected (Supplementary Figure 2B) and background signal was isolated from the scaffold with a thresholding step. Then, a particle analysis was run to count the number of “pores” ($n_{measured}$) and measure their area ($A_{measured}\left( i \right)$), where $i$ indicates a specific pore in the range of $\left[ i=1\ldots n_{measured} \right]$. The pore ratio ($r$) was determined by,

|  | $r= \frac{n_{measured}}{n_{design}}$ | (5) |
| --- | --- | --- |

where $n_{design}$ indicates the number of pores and $A_{design}\left( i \right)$ their respective area, that were designed. The pore ratio was validated against a second quality measure “open surface” ($Q$) determined as,

|  | $Q= \frac{\sum_{i=1}^{n_{measured}} A_{measured}\left( i \right)}{\sum_{i=1}^{n_{design}} A_{design}(i)}\cdot100$ | (6) |
| --- | --- | --- |

A value of $Q=100\%$ corresponds to a printed scaffold that conforms to the theoretical design (no fibre misalignment), while values lower than 100% indicate a shift in fibre stacking. Values $Q=0\%$ indicate that the fibres are randomly distributed covering the entire open surface. Since no significant differences between pore ratio and open surface were found, only results for pore ratio were presented.

**Chondrogenic differentiation medium**: Chondrogenic differentiation medium consisted of Dulbecco’s modified eagle medium (31966, Thermo Fisher Scientific, USA) supplemented with 1% penicillin/streptomycin, 1% l-ascorbic acid-2-phosphate, 1% ITS + Premix Universal culture supplement (Corning, USA), 2.5% HEPES (1M, Gibco, Thermo Fisher Scientific, USA), 0.4% dexamethasone (0.1 x 10^-6^ M, Sigma Aldrich, USA) and 0.1% recombinant human transforming growth factor-β1 (TGF-β1) (10 ng/mL, Prepotech, UK). Medium was refreshed three times per week.

**Supplementary data and figures**

**Supplementary Table 1.** Surface roughness of investigated materials

| **Substrate biomaterial** | **Average roughness (R_a,_ µm)** | **Route mean square roughness (R_q ,_ µm)** |
| --- | --- | --- |
| Polycaprolactone (PCL) | 0.21 | 0.32 |
| Magnesium phosphate-based cement (MgP) | 4.41 | 5.21 |
| Gelatine methacryloyl (gelMA) | 3.64 | 4.50 |
| Aluminium (Al, control) | 0.07 | 0.09 |


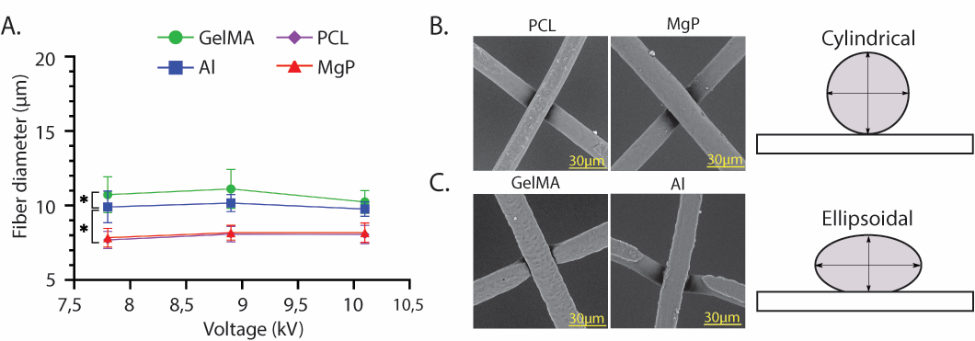


**Supplementary Figure 1.** A) Effect of applied voltage on fibre diameter. * = p < 0.05. Representative SEM images and schematic illustration of B) cylindrical fibres collected onto PCL and MgP substrates and C) ellipsoidal fibers collected onto gelMA and Al.

**
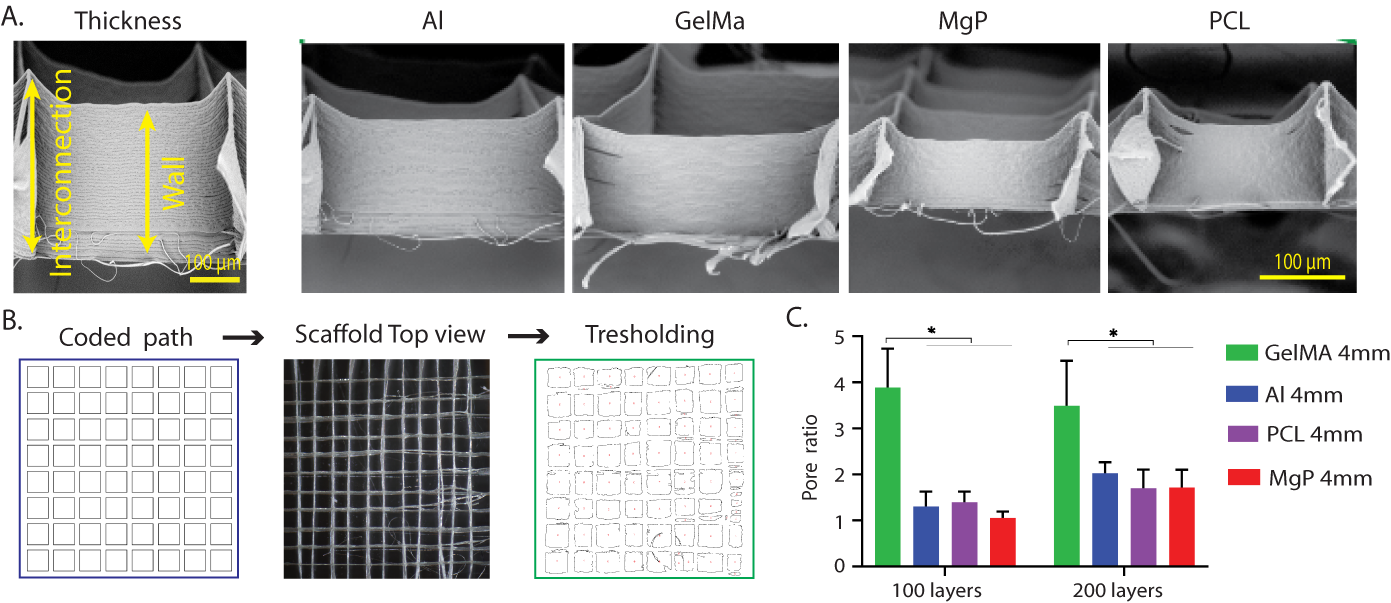
**

**Supplementary Figure 2.** A) Representation of measured differences between interconnection and wall thickness and representative SEM images of MEW scaffolds cross section collected onto different substrate materials. B) Imaging methodology used to quantify pore ratio. Top view images of coded path, printed scaffold and respective thresholding. C) Variation of pore ratio of scaffolds deposited on 4 mm thick collectors (r = 1 indicates a printed scaffold that conforms to the planned design, while values r > 1 indicates imperfect fibre stacking). * = p < 0.05.


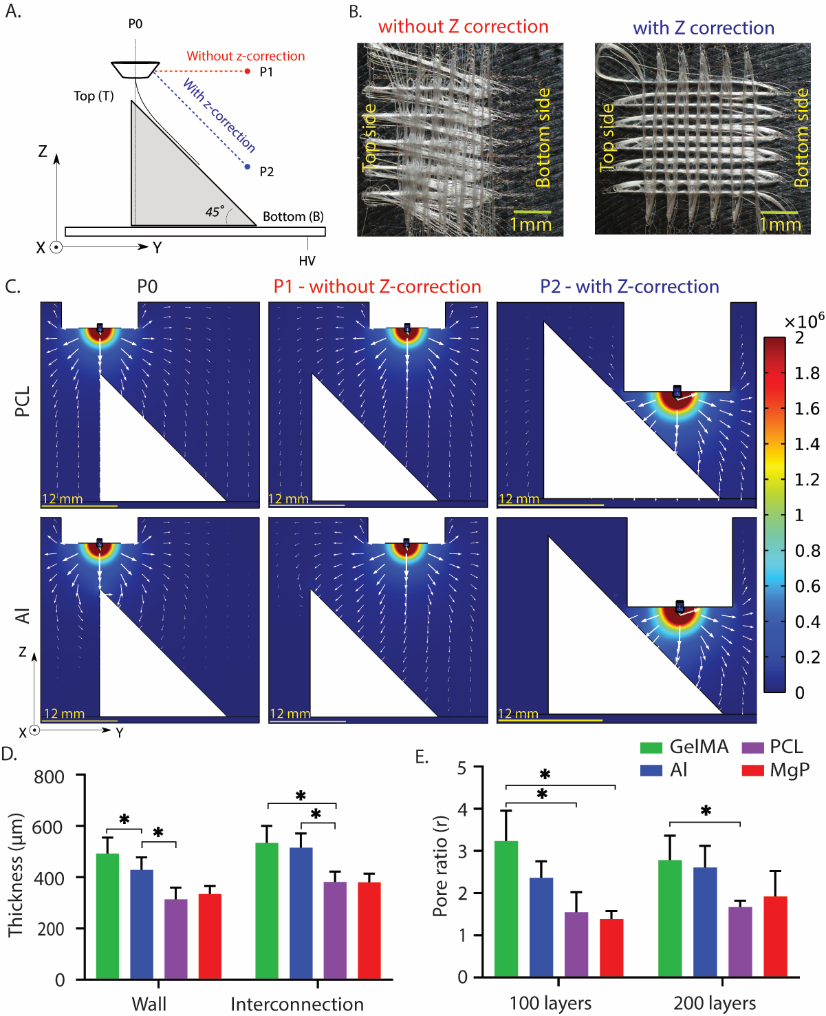


**Supplementary Figure 3**: **Fibre collection on wedge-shaped collecting materials (PCL, MgP, gelMA, and Al).** A) Schematic representation of the evaluated printhead trajectories with and without z-correction. B) Representative stereoscopic images of scaffolds printed on aluminium wedge-shaped structures with and without z-correction of the printhead trajectory. C) Computational simulation of EF strength (V/m) and distribution (white arrows in logarithmic scale) for a non-conductive (PCL) and conductive (aluminium) wedge-shaped collecting materials. E) Quantification of final scaffold thickness at the walls and interconnections of the deposited fibres and of the pore ratio of scaffolds with 100 and 200 μm fibre spacing deposited on wedge-shaped collecting materials with z-correction in the printhead trajectory. * = p < 0.05

**
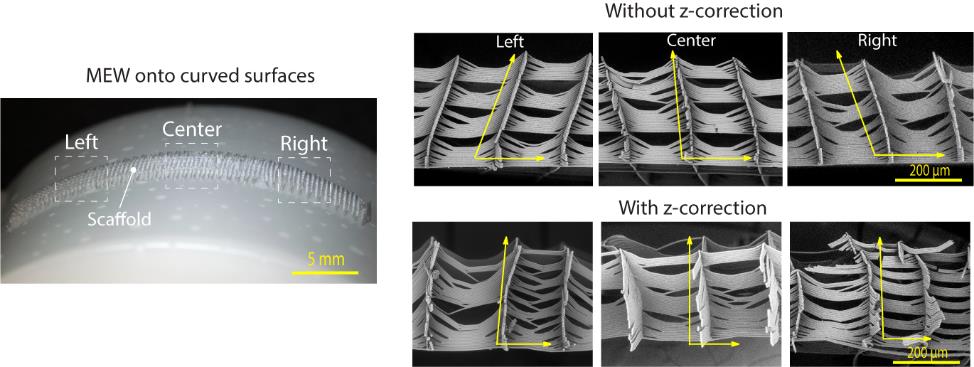
**

**Supplementary Figure 4.** Representation stereoscopic image of melt electrowritten PCL scaffolds onto a curved PCL substrate with and without z-correction. Representative SEM detailing the microstructure deformation at center, left and right (lateral) part of a PCL curved substrate. Yellow arrows highlight out-of-plane scaffold deformation.


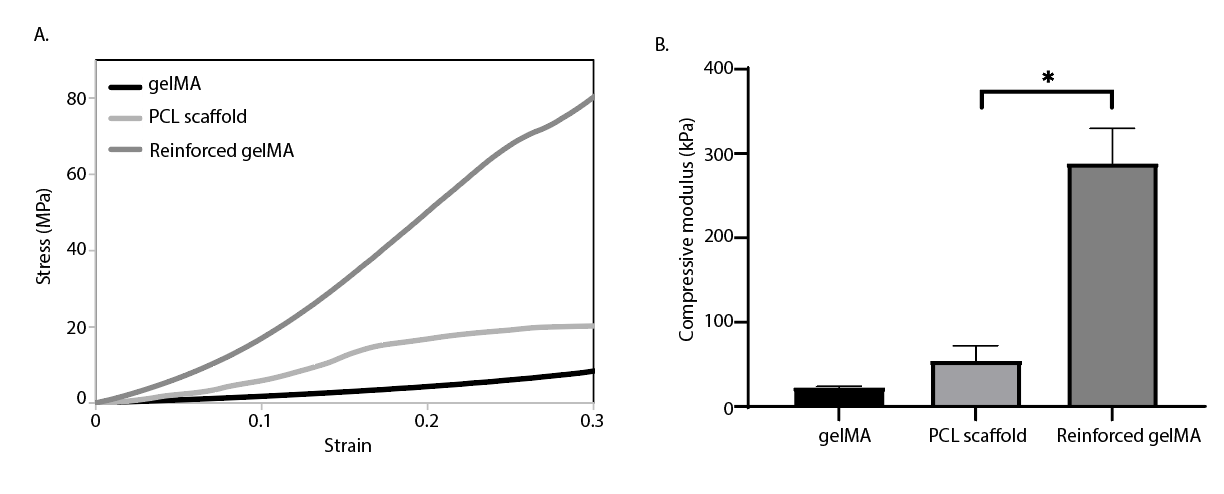


**Supplementary Figure 5. Reinforcement effect of melt electrowritten PCL scaffolds incorporated in gelMA hydrogel.** These PCL scaffolds show the same inter fibre spacing (400 µm) as compared to the ones that are used in the proof-of-principle *in vitro* study. A) representative stress-strain curves. B) compressive modulus of gelMA only, fibre reinforcing PCL scaffold only, and reinforced gelMA.

**Supplementary Movies**

**Supplementary Movie 1:** Melt electrospinning writing on a wedge-shaped substrate. The printhead trajectory in z-direction follows the shape of the ceramic non-flat, wedge substrate.

**Supplementary Movie 2:** Melt electrospinning writing on a dome-shaped substrate. The printhead trajectory in z-direction follows the shape of the non-flat, hydrogel, dome-shaped substrate.
